# Omnivore-herbivore interactions: thrips and whiteflies compete via the shared host plant

**DOI:** 10.1038/s41598-018-22353-2

**Published:** 2018-03-05

**Authors:** Maria L. Pappas, Georgia Tavlaki, Anneta Triantafyllou, George Broufas

**Affiliations:** Democritus University of Thrace, School of Agricultural and Forestry Sciences, Department of Agricultural Development, Orestiada, 68200 Greece

## Abstract

Phytophagy is a common feature among pure herbivorous insects and omnivores that utilise both plant and prey as food resources; nevertheless, experimental evidence for factors affecting their interactions is restricted to intraguild predation and predator-mediated competition. We herein focused on plant-mediated effects that could result from plant defence activation or quality alteration and compared the performance of an omnivore, the western flower thrips *Frankliniella occidentalis*, and a pure herbivore, the greenhouse whitefly *Trialeurodes vaporariorum*, on cucumber plants previously infested with either species. Furthermore, we recorded their behavioural responses when given a choice among infested and clean plants. Whiteflies laid less eggs on plants previously exposed to thrips but more on whitefly-infested plants. Thrips survival was negatively affected on whitefly-infested than on thrips-infested or clean plants. Notably, whiteflies developed significantly faster on plants infested with conspecifics. In accordance, whiteflies avoided thrips-infested plants and preferred whitefly-infested over clean plants. Thrips showed no preference for either infested or clean plants. Our study is a first report on the role of plant-mediated effects in shaping omnivore-herbivore interactions. Considering the factors driving such interactions we will likely better understand the ecology of the more complex relationships among plants and pest organisms.

## Introduction

Unable to run away from their enemies, plants are often exposed to a variety of phytophagous arthropods. Interactions among herbivores sharing the same host plant include direct interference, exploitative competition, apparent competition or mutualism and plant-mediated effects^1^. These interactions have been shown to be common and highly asymmetrical, not related to the amount of the plant tissue removed, resource partitioning in space and time or the feeding guild of the interacting species^2^.

Among co-occurring phytophagous arthropods, omnivores are capable of exploiting both animal and plant food. This behaviour is advantageous because of the fitness benefits of diet mixing for omnivores, also ensuring survival in the absence of animal prey^3^. For example, the western flower thrips *Frankliniella occidentalis*, which is a major pest in several crops is also known to feed on herbivores such as spider mites and whiteflies^4–6^. This mixed diet on both plants and prey results in increased fecundity and longevity of thrips compared to a diet on leaf tissue only^5,7^. Therefore, herbivores that co-exist with omnivores on the same plant should not only have to compete for the shared food resource (exploitative competition for the plant) but also face omnivore predation (direct interference or intraguild predation).

Apart from directly affecting herbivores via predation, omnivores have been shown to engage in indirect interactions such as apparent competition and apparent mutualism with pure herbivores. In general, apparent competition is based on the assumption that the numerical response of a shared natural enemy’s population will increase in the presence of two or more prey species, and this increase will negatively affect one or all the interacting herbivores^8,9^. In contrast, the satiation of the shared predator may result in reduced consumption of either prey and thus to apparent mutualism among the interacting species^9,10^. Both types of indirect interactions have been shown for omnivores and herbivores that co-occur in agricultural settings. For example, in the short term apparent mutualism was shown for *F*. *occidentalis* and *T*. *vaporariorum* in the presence of the predatory mite *Amblyseius swirskii*^11^, whereas the same arthropods plus the spider mite *Tetranychus urticae* in the long-term engaged in apparent competition resulting in improved whitefly and spider mite control^12^. Enhanced biological control in the above studies was attributed to predator facilitation in the presence of more than one pest species however without considering intraguild predation and/or plant-mediated interactions among the competing herbivores.

In response to herbivory, plants may affect indirect interactions between pure herbivores through the induction of defences and/or via changes in plant tissue quality^13,14^. These plant traits are known to directly or indirectly influence the performance (e.g. development, fecundity) and/or behaviour (e.g. host preference) of herbivores^13,15^. Although plant-mediated interactions among omnivores and herbivores have often been assumed^6,16,17^, the ways these organisms interact through the plant have only been indirectly addressed so far. Plant quality has been shown to affect intraguild predation intensity and/or diet selection decisions by omnivores^18–21^ in a prey species-related manner. For example, adult thrips consumed more spider mite eggs on sweet pepper (poor quality host) than on cucumber (high quality host) plants^20^ whereas spider mite damage on cucumber plants resulted in thrips shifting from plant- to prey-feeding^6^. In contrast, thrips predation rate was shown not to be related to host plant quality in the presence of whitefly prey^5^. Both omnivores and pure herbivores are capable of inducing plant defence responses and/or altering plant quality through their phytophagy^13,15,22^. However, to the best of our knowledge, bi-directional effects of plant-mediated responses on the performance and behaviour of these interacting arthropods have not been addressed so far. The fact that pure herbivores only feed with the plant whereas omnivores exploit both plant and animal food suggests a differential reliance of these organisms on plants as food source and a stronger intimacy of herbivores with plants. Therefore, omnivores and herbivores may have evolved different mechanisms to tolerate plant responses to their own or other arthropods phytophagy.

**Figure 1:**
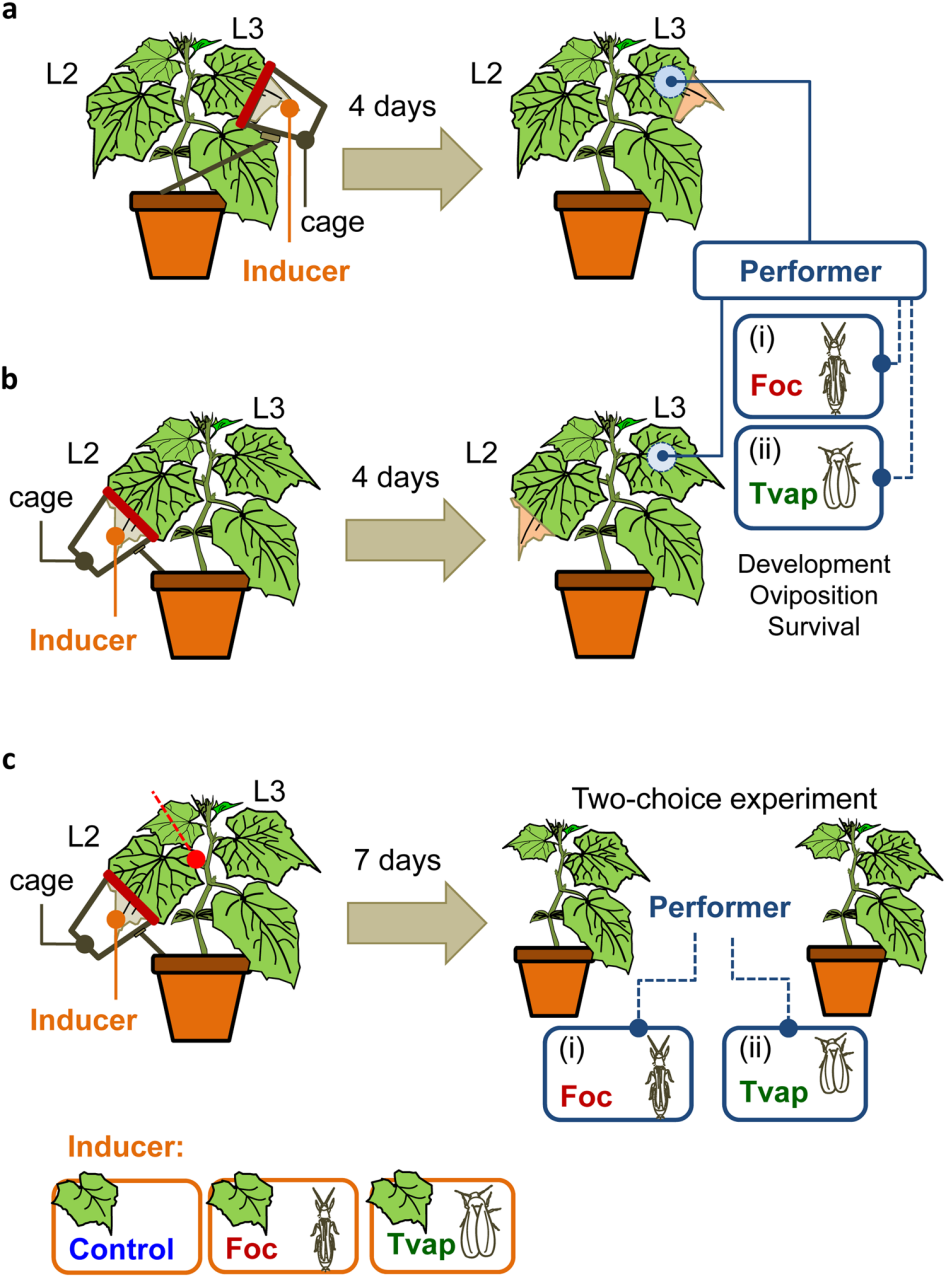
Overview of the experimental design and set-ups used in this study. Experimental treatments used to assess the local (**a**) and systemic (**b**) effects of prior infestation of cucumber plants with whiteflies or thrips on their oviposition, survival and development. Preference was assessed in two-choice greenhouse experiments (**c**). ‘Tvap’, whitefly, *Trialeurodes vaporariorum*; ‘Foc’, thrips, *Frankliniella occidentalis*; ‘L2’, 2^nd^ oldest leaf; ‘L3’, 3^rd^ oldest leaf; ‘Control’, no insects. For more details about the different treatments and set-ups, refer to the Methods section.

We hypothesized that omnivores and pure herbivores interact through the plant and that plant-mediated effects are reflected on their performance and/or behaviour. Our experimental system consisted of the omnivore thrips *F*. *occidentalis* and the herbivorous whitefly *T*. *vaporariorum*. These two species overlap in the field and, as noted above, are known to interact directly through intraguild predation^5^, but also indirectly via apparent mutualism/competition^11,12^. We herein further argue that these species interact via plant-mediated effects. We tested bi-directional local and systemic effects of exposing cucumber plants on each herbivore on the performance of the other species (Fig. 1). To measure the impacts of plant-mediated effects only intraguild predation was excluded by temporally and spatially separating thrips and whiteflies. Since we found evidence for plant-mediated competition, we further assessed the extent to which host-preference decisions of the competing omnivores and herbivores are determined by plant-mediated effects (Fig. 1).

## Results

To unravel the role of plant-mediated effects in shaping whitefly-thrips interactions, we exposed cucumber plants on either species and then recorded each species development, survival and oviposition on exposed and unexposed (clean) plants. Whiteflies and thrips preference for all combinations of treated and clean plants was assessed in greenhouse release-recapture experiments.

### Whitefly performance

**Figure 2:**
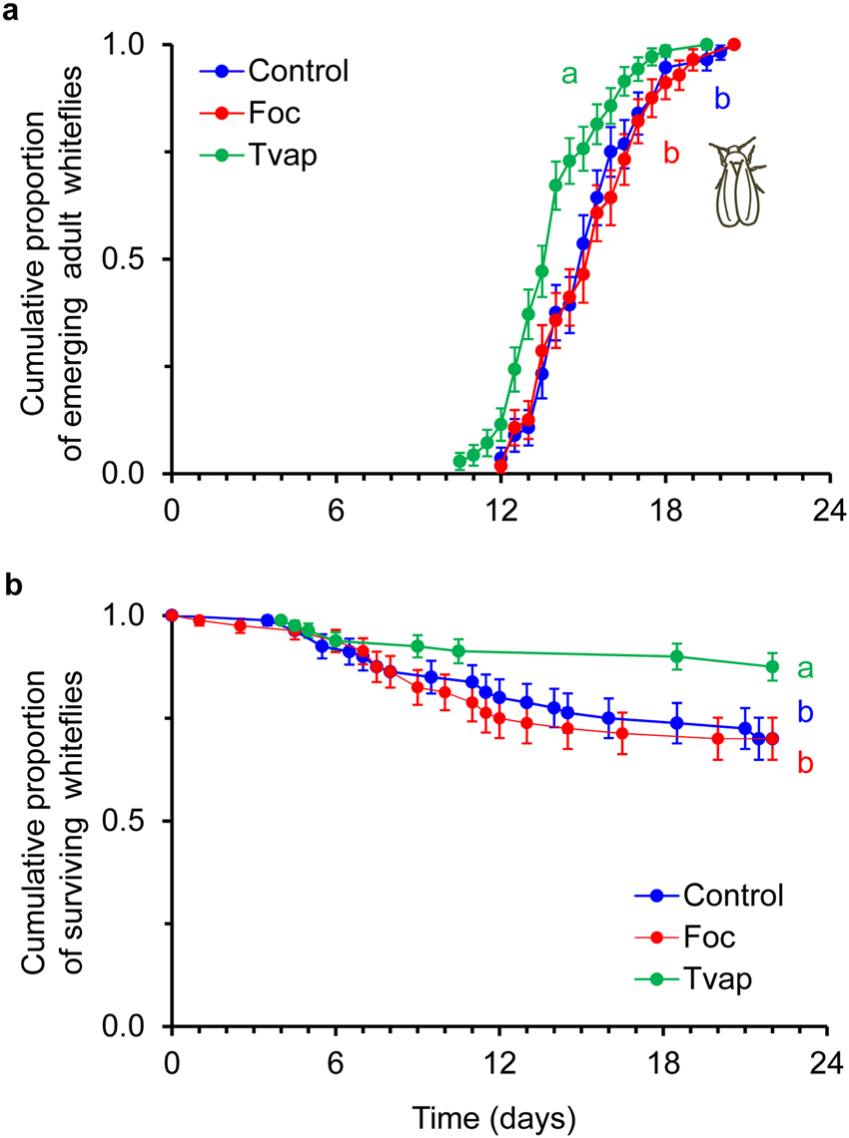
Effects of prior infestation of cucumber plants with whiteflies or thrips on whitefly development. Three to four week old plants (n = 16) were infested with 20 female whiteflies or thrips on the second oldest leaf (L2) for 4 days. Control plants received no insects. Whitefly crawlers were transferred on leaf discs (n = 5 per plant) that were cut out the L3 leaf (systemic effects). Shown are (**a**) the cumulative proportions of juveniles that developed into adults (±s.e.) and (**b**) the cumulative survivorship of whiteflies (±s.e.) on whitefly-infested (Tvap, green line), thrips-infested (Foc, red line) and control (Control, blue line) plants. Per panel, lines with different letters are significantly different (Tukey multiple comparisons after applying a Cox proportional hazards model, *P* < 0.05).

Whiteflies reached adulthood significantly faster on plants infested with adult whiteflies than on thrips-infested or clean plants where whitefly developmental rate was similar (Fig. 2, Table 1). Juvenile survival was significantly higher on whitefly-infested plants than on thrips-infested and clean plants (Fig. 2, Table 1).

**Table 1:** Results of statistical tests of performance parameters of whiteflies and thrips on whitefly-infested, thrips-infested or control plants. Values of *P* < 0.05 are given in bold. All degrees of freedom are 2. ^a^Cox proportional hazards mixed effects, value is Chisq. ^b^Generalized mixed effects model with a Poisson error distribution, value is Chisq. ^c^Generalized linear model with a binomial error distribution, value is deviance.

Performance parameters	Value	*P*
**Whiteflies**		
Juvenile survival^a^	10.127	**0.0063**
Developmental time^a^	17.291	**0.0001**
Oviposition (local, day 4)^b^	610.02	**<0.001**
Adult survival (local, day 4)^c^	0.44	0.8040
Oviposition (systemic, day 4)^b^	105.01	**<0.001**
Adult survival (systemic, day 4)^c^	0.55	0.7603
**Thrips**		
Juvenile survival^a^	14.91	**0.0006**
Developmental time^a^	2.39	0.3020
Dispersal^a^	1.05	0.5899
Oviposition (local, day 4)^b^	2.85	0.2406
Adult survival (local, day 4)^c^	0.63	0.7302
Oviposition (systemic, day 4)^b^	1.69	0.4293
Adult survival (systemic, day 4)^c^	0.51	0.7761

**Figure 3:**
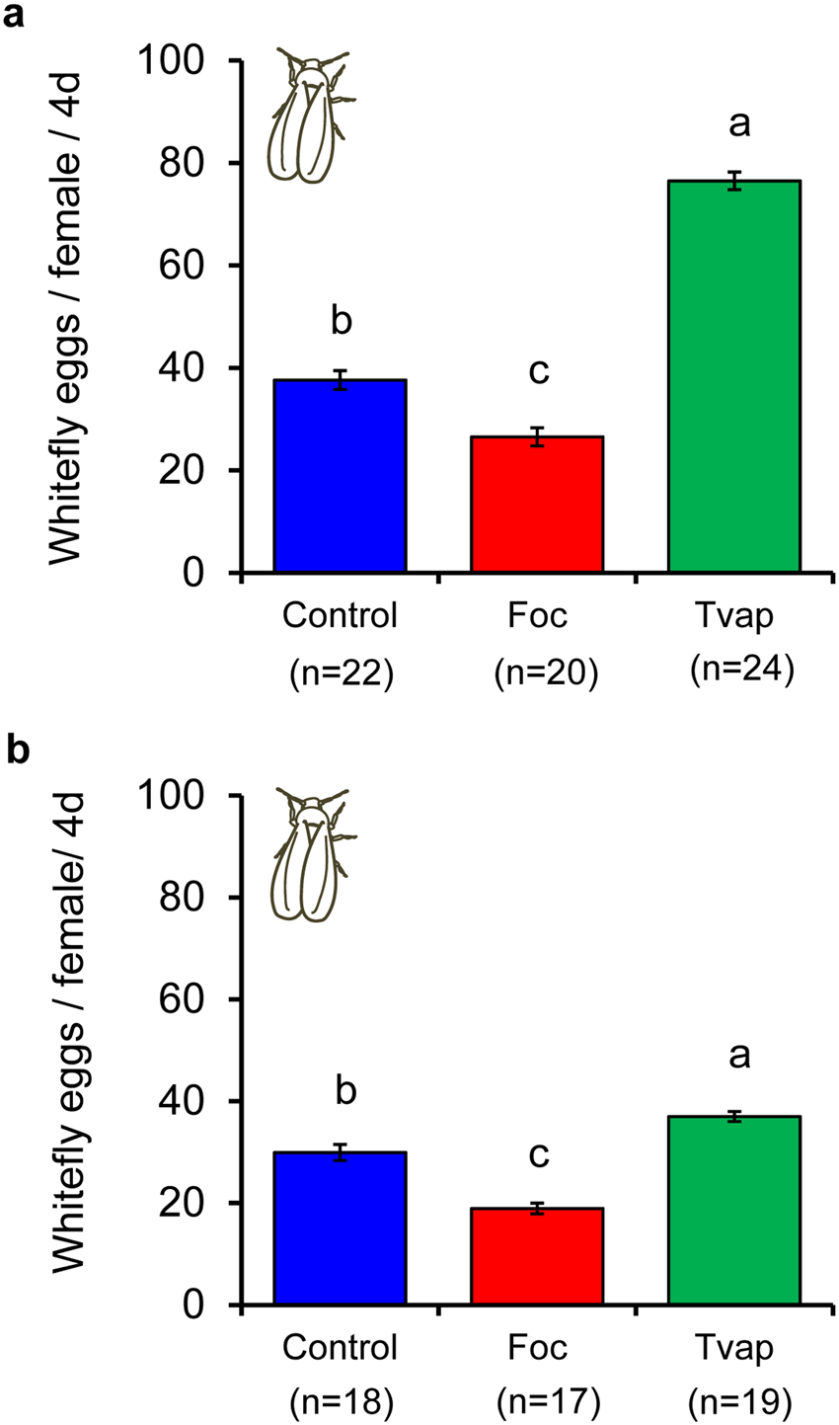
Effect of prior infestation of cucumber plants with whiteflies or thrips on whitefly oviposition. Three to four week old plants were infested with 20 female whiteflies or thrips on the L3 leaf (local effects experiment) or the 2^nd^ (L2) oldest (systemic effects experiment) for 4 days. Control plants received no insects. One whitefly female (7–10 days old) was enclosed in a clip cage attached on the abaxial surface of L3 leaf. Shown are the average number of eggs (±s.e.) per plant laid by each female during 4 days on (**a**) the exposed L3 leaf (local effects experiment) and (**b**) the unexposed L3 leaf (systemic effects experiment) of whitefly-infested (Tvap, green bar), thrips-infested (Foc, red bar) and control (Control, blue bar) plants. Numbers inside brackets are numbers of replicates (plants). Per panel, bars with different letters are significantly different (Tukey multiple comparisons after applying a generalized linear mixed model with a Poisson error distribution, *P* < 0.001).

Whitefly oviposition on locally treated plants was significantly affected by treatment (Fig. 3, Table 1). Prior infestation of plants with thrips resulted in a 27% decrease in the number of eggs laid during the subsequent 4 days on the same leaf (L3) where infestation took place relative to control plants. Notably, eggs laid on whitefly-infested plants was double the number recorded on clean plants (Fig. 3).

Similarly, there was a significant effect of treatment on whitefly oviposition on systemically treated plants (Fig. 3, Table 1). Oviposition on leaf L3 was positively affected by previous infestation of leaf L2 with whiteflies and negatively affected on thrips-infested plants (Fig. 3).

Adult whitefly survival remained unaffected and ranged between 80 and 90% for locally and systemically treated plants, respectively (Table 1).

### Thrips performance

**Figure 4:**
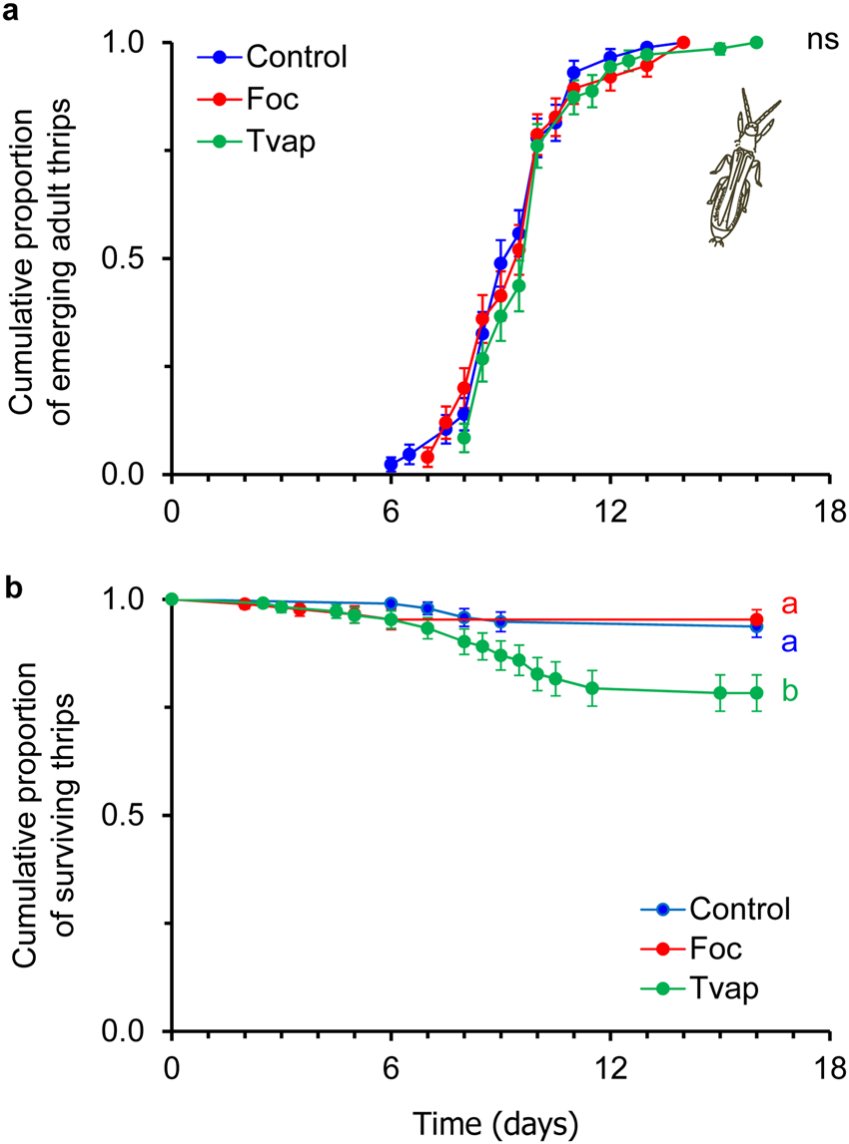
Effect of prior infestation of cucumber plants with whiteflies or thrips on thrips development. Three to four week old plants (n = 24) were infested with 20 female whiteflies or 20 thrips on the second oldest leaf (L2) for 4 days. Control plants received no insects. First instar thrips larvae were transferred on leaf discs (n = 5 per plant) that were cut out the L3 leaf (systemic effects). Shown are (**a**) the cumulative proportions of juveniles that developed into adults (±s.e.) and (**b**) the cumulative survivorship of whiteflies (±s.e.) on whitefly-infested (Tvap, green line), thrips-infested (Foc, red line) and control (Control, blue line) plants. There were no significant differences in the developmental rate among treatments. Lines with different letters in panel (**b**) are significantly different (Tukey multiple comparisons after applying a Cox proportional hazards model, *P* < 0.05).

There was no significant effect of treatment on thrips developmental rate and dispersal (Fig. 4, Table 1). Juvenile survival was significantly lower on whitefly-infested plants than on thrips-infested or control plants (Fig. 4, Table 1).

**Figure 5:**
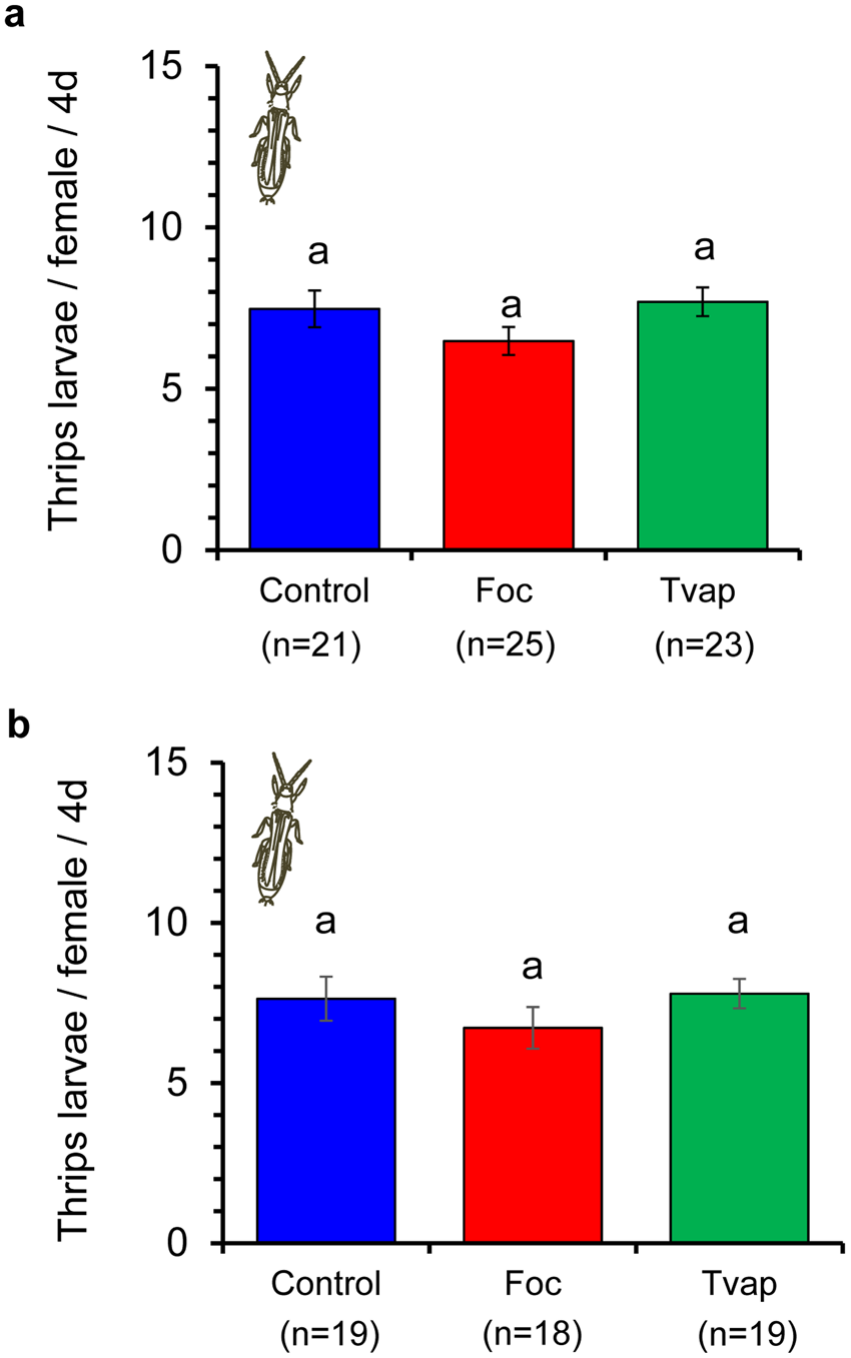
Effect of prior infestation of cucumber plants with whiteflies or thrips on thrips fecundity. Three to four week old plants were infested with 20 female whiteflies or thrips on the L3 leaf (local effects experiment) or the 2^nd^ (L2) oldest (systemic effects experiment) for 4 days. Control plants received no insects. One thrips female (7–10 days old) was enclosed in a clip cage attached on the abaxial surface of L3 leaf. Shown are the average number of hatching larvae (±s.e.) per plant laid by each female during 4 days on (**a**) the exposed L3 leaf (local effects experiment) and (**b**) the unexposed L3 leaf (systemic effects experiment) of whitefly-infested (Tvap, green bar), thrips-infested (Foc, red bar) and control (Control, blue bar) plants. Numbers inside brackets are numbers of replicates (plants). There were no significant differences in fecundity among treatments.

There was no significant effect of treatment on thrips fecundity and survival on locally or systemically treated plants (Fig. 5, Table 1).

### Whitefly and thrips preference

**Figure 6:**
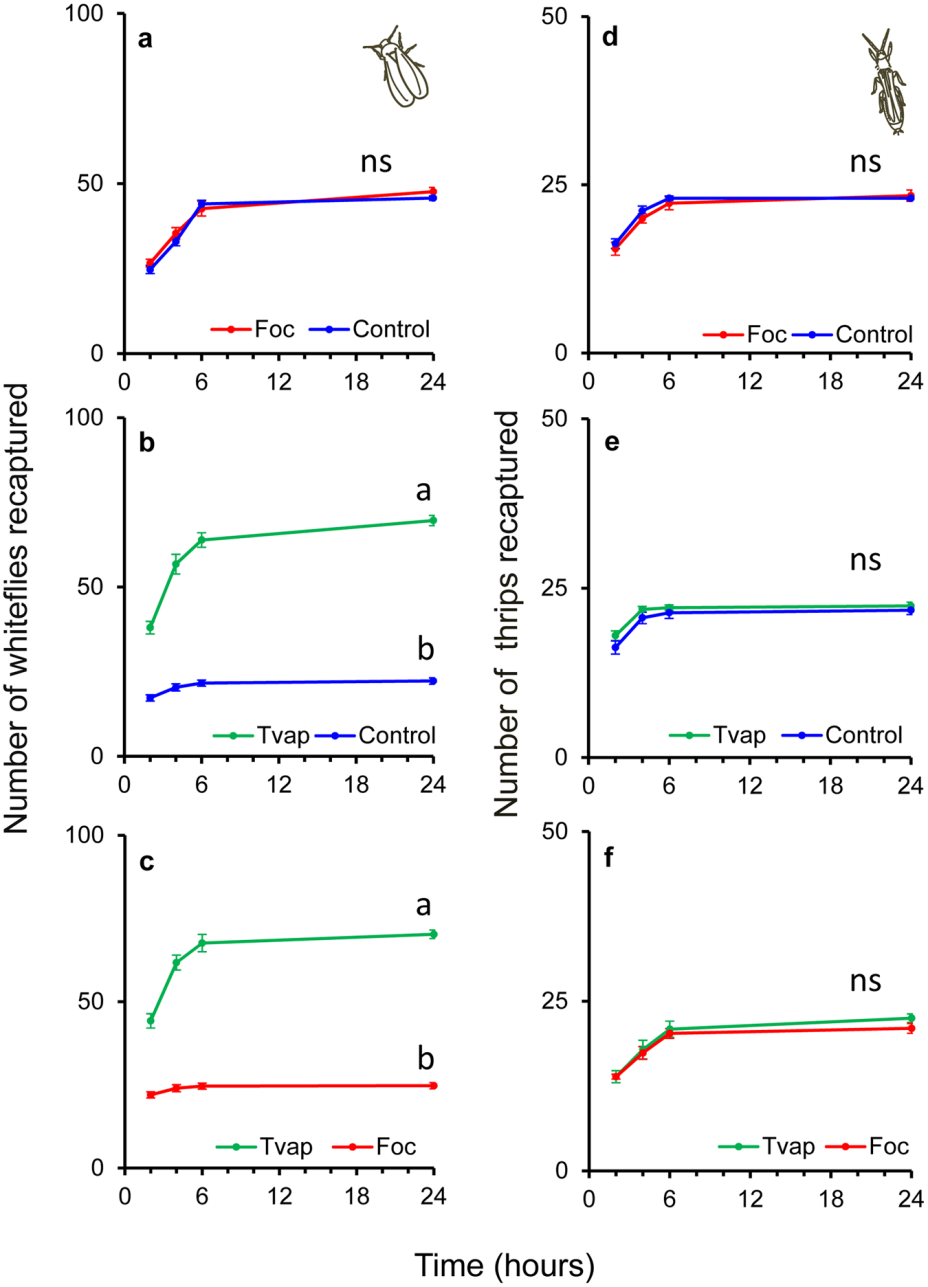
Effect of prior infestation of cucumber plants with whiteflies or thrips on whitefly and thrips preference in the greenhouse (two-choice experiments). Three to four week old plants were infested with 20 female thrips or 50 whiteflies per plant on the 2nd (L2) oldest leaf for 7 days. L2 leaf was cut from all plants on day 8 and 100 whiteflies (**a**–**c**) or 50 thrips (**d**–**f**) were allowed to choose among thrips-infested (red line) over control plants (blue line), whitefly-infested (green line) over control plants (blue line) and whitefly-infested (green line) over thrips-infested plants (red line) (n = 4 plant pairs per treatment and species). Shown are the numbers (±s.e.) of insects recaptured on the plants as a function of time (recordings took place 2, 4, 6 and 24 hours after whitefly/thrips release, refer to the Methods section for further details). Per panel, lines with different letters indicate significant differences in the distribution over the two plants (infestation types) after applying a generalized linear mixed model with a Poisson error distribution, *P* < 0.001; ns, not significant).

There was a significant overall effect of choices available and time on the preference of whiteflies for plants of each infestation type (Table 2). However, the interaction of choices with time had no significant effect on whitefly preference. This was caused by the proportions of whiteflies landing on plants gradually increasing through time in a similar way among different choice-tests (Table 2). Whitefly distribution among whitefly-infested plants and thrips-infested or clean plants was significantly affected by the interaction of infestation type and time (Fig. 6b,c, Table 3). This was due to a gradual increase of the number of whiteflies on whitefly-infested plants, but not on thrips-infested or clean plants (Fig. 6b,c). In contrast, the distribution of whiteflies did not change with time and was not significantly different among thrips-infested and clean plants (Fig. 6a, Table 3).

**Table 2:** Overall statistics of preference of whiteflies and thrips in two-choice greenhouse experiments. Results are from generalized mixed effects models with a binomial error distribution. Values of *P* < 0.05 are given in bold.

Whiteflies	d.f.	Chisq	*P*
Choice (combination of plants)	2	418.32	**<0.001**
Time	3	12.44	0.0063
Treatment * Time	6	9.69	0.1381
**Thrips**	**d**.**f**.	**Chisq**	***P***
Choice (combination of plants)	2	0.18	0.9125
Time	3	0.73	0.8652
Treatment*Time	6	2.51	0.8674

**Table 3:** Results of statistical tests of the distribution of whiteflies and thrips over the two plants (each representing a different infestation type) of each choice combination through time in two-choice greenhouse experiments. Results are from generalized mixed effects models with a Poisson error distribution. Values of *P* < 0.05 are given in bold.

Whiteflies	d.f.	Chisq	*P*
**Thrips-infested vs**. **Clean plants**			
Infestation type	1	1.44	0.2304
Time	3	118.19	**<0.001**
Infestation type * Time	3	0.21	0.9760
**Whitefly-infested vs**. **Clean plants**			
Infestation type	1	667.87	**<0.001**
Time	3	107.37	**<0.001**
Infestation type * Time	3	12.04	**0.0085**
**Whitefly-infested vs**. **Thrips-infested plants**			
Infestation type	1	488.18	**<0.001**
Time	3	48.43	**<0.001**
Infestation type * Time	3	9.88	**0.0196**
**Thrips**	**d**.**f**.	**Chisq**	***P***
**Thrips-infested vs**. **Clean plants**			
Infestation type	1	0.06	0.8048
Time	3	27.29	**<0.001**
Infestation type * Time	3	0.37	0.9453
**Whitefly-infested vs**. **Clean plants**			
Infestation type	1	1.039	0.3081
Time	3	12.56	**0.0057**
Infestation type * Time	3	0.22	0.9736
**Whitefly-infested vs**. **Thrips-infested plants**			
Infestation type	1	0.61	0.4334
Time	3	32.90	**<0.001**
Infestation type * Time	3	0.21	0.9747

The overall preference of thrips for plants of each infestation type was not affected by the choices available, the time or their interaction (Table 2). Within each choice test, the interaction between infestation type and time had no significant effect on the distribution of thrips among the two plants but there was a significant effect of time that was caused by the increasing number of thrips landing on plants irrespectively of the infestation type.

## Methods

### Plants and Insects

Cucumber (*Cucumis sativus* L., cv Ginga F1, Geostore SA) plants were grown from seed in plastic pots (Ø 12 cm) in a climate room (25 ± 2 °C, 16:8 LD, 60–70% RH). They were watered every other day and fertilized once a week (N-P-K, 20–20–20). The plants received no pesticide treatment.

Whiteflies (*Trialeurodes vaporariorum*) were collected from greenhouse cucumber in Alexandria (Greece) and were reared since 2011 at the Department of Agricultural Development at the Democritus University of Thrace on cucumber plants (cv Ginga F1, Geostore SA) in plastic cages (47.5 × 47.5 × 93.0 cm, type 44590F, BugDorm) at 25 ± 2 °C, 16:8LD. Plants used for insect rearing were 4 to 5 weeks old. To obtain young (7–10 days old) females, clean plants were infested with whitefly females and allowed to lay eggs for 24 hours. Plants were 3 to 4 weeks old when used for the experiments.

Thrips (*Frankliniella occidentalis*) were originally collected in 2008 from *Chrysanthemum* sp. plants in Orestiada (Greece). Since then, the rearing was maintained at the Department of Agricultural Development at the Democritus University of Thrace on cucumber leaves placed on wet cotton wool in Petri dishes (14 cm diameter) in a climate room (25 ± 2 °C, 16:8 LD, 60–70% RH). Cattail (*Typha latifolia*) pollen was added on the leaves twice a week. Young (7–10 days old) females were obtained by allowing female thrips to oviposit for 24 hours on cucumber leaves.

### Performance experiments

To assess the effects of prior infestation with either herbivore (thrips or whitefly) on the performance of the forthcoming herbivore (whitefly or thrips, respectively), we followed a common experimental setup for both species (Fig. 1). Three to four weeks old cucumber plants (4th leaf fully expanded and emerging 5th leaf) were infested with a high number of the ‘Inducer’ species for a period of 4 days. For the infestation phase, transparent plastic cups (750 ml) were adjusted at the tip of the leaf (Fig. 1). An opening on the lid of the cup (8 cm length, 2 cm width) was used to introduce approx. one third of each leaf inside each cup without damaging the leaf. A thin lanolin paste layer was applied parallel to both sides of the opening to ensure no space would allow insects to escape. ‘Inducers’ were transferred in each cup through a small circular opening on the opposite side of the cup which was then closed with cotton wool. The cups were safely maintained on the plant by adjusting them on wooden sticks (30 cm in length). At the end of the infestation period, the insects were removed and one female for each ‘Performer’ species was transferred on the same or the next younger leaf depending on the treatment (local or systemic effect, respectively). The survival and oviposition of each female was recorded after a subsequent period of 4 days. In a separate group of experiments, the cup and insects were removed and juvenile development and survival of each insect species were recorded twice daily at 12-hour intervals until adulthood. For each treatment (i.e. Inducer-Performer species combination), control plants received an empty cup for the course of the infestation period (4 days) (Fig. 1). All experiments were conducted at 25 ± 2 °C, 16:8 LD, 60–70% RH.

### Whitefly oviposition and survival

To test for local effects of prior infestation of plants with thrips or whiteflies on whitefly performance, 20 young (7–10 days old) thrips or whitefly females were introduced to plastic cups attached on the tip of the 3^rd^ leaf, as described above. After 4 days, the cups and the insects were removed and one clip-cage (2.5 cm in diameter) was attached on the abaxial surface of the same (3^rd^) leaf, but in a distance of 1 cm from the infested tip (Fig. 1). One young female adult (7–10 days old) was introduced in each clip cage and allowed to feed and lay eggs for 4 days.

Systemic effects on whitefly performance were tested by infesting cucumber plants on the 2^nd^ leaf with 20 young thrips or whitefly females, as described above for the local effects experiments. After 4 days, the cups and the insects were removed and one clip-cage was attached on the abaxial surface of the next (3^rd^) leaf (Fig. 1).

For both experiments, survival and oviposition of each female were recorded on the 4^th^ day with the use of a binocular. Experiments were repeated in two time blocks with 20–24 and 17–19 plants per treatment for local and systemic effects, respectively. Differences in the number of whitefly eggs were analysed with a generalized linear mixed model (GLMM, function ‘glmer’ in R package ‘lme4’^23^) with a Poisson error distribution. In this model, treatment was specified as fixed factor and replicate (plant) nested within block in time as random factor. The proportions of live whiteflies were analysed with a generalized lineal model (GLM, function ‘glm’ in R package ‘lme4’^23^) with treatment as fixed factor and a binomial error distribution. When significant differences were found, multiple comparisons were performed using Tukey contrasts (function ‘ghlt’ in R package ‘multcomp’^24^). All statistical analyses were performed with the R software, version 3.2.5^25^.

### Thrips fecundity and survival

Local and systemic effects of previous infestation of plants on forthcoming thrips performance were tested as described above for whiteflies. Because thrips eggs are not visible (females insert their eggs in the plant tissue), we only recorded larval hatching with the use of a binocular as an indirect estimate of thrips oviposition. On the 4^th^ day after removing the cups and the insects, thrips survival was recorded and leaf discs (3 cm diameter) were punched out the area that was restricted inside the clip cage. The discs were placed upside down on a wet cotton wool in plastic Petri dishes (5.5 cm diameter) in a climate box (25 ± 2 °C, 16:8 LD, 60–70% RH). For the following 10 days, the leaf discs were daily inspected and the number of hatched larvae was recorded. Experiments was repeated in two time blocks with 21–25 plants and 18–19 plants per treatment for local and systemic effects, respectively. Differences in the number of hatching thrips larvae were analysed with a generalized linear mixed model (GLMM, function ‘glmer’ in R package ‘lme4’^23^) with a Poisson error distribution. In this model, treatment was specified as fixed factor and replicate (plant) nested within block in time as random factor. The proportions of live thrips were analysed with a generalized linear model (GLM, function ‘glm’ in R package ‘lme4’^23^) with treatment as fixed factor and a binomial error distribution.

### Juvenile development and survival

To test for plant-mediated effects on whitefly or thrips development and survival, cucumber plants were infested on the 2^nd^ leaf with 20 young thrips or whitefly females for a period of 4 days as described above in a separate group of experiments. Afterwards, leaf discs (3 cm in diameter) were punched out the next (3^rd^) younger leaf using a hole punch and were placed on wet cotton wool in Petri dishes (5 leaf discs per plant). One whitefly crawler (younger than 24 hours old) or a newly hatched thrips larva was transferred on each leaf disc and subsequently the survival and development of each individual was recorded every 12 hours until adulthood. This experiment was performed in two time blocks with 16 plants per treatment for whiteflies and in three time blocks with 24 plants per treatment for thrips. Juvenile development, survival and dispersal (in the case of thrips) were analysed with a time-to-event analysis (Cox proportional hazards model, R package ‘coxme’^26^) with treatment as fixed factor and replicate (leaf disc) and plant nested within block as random factors. In the case of significant differences, multiple comparisons were performed using Tukey contrasts as above.

### Greenhouse two-choice experiments

Thrips and whitefly preference was assessed for (1) whitefly-infested over clean plants, (2) whitefly-infested over thrips-infested plants and (3) thrips-infested over clean plants in parallel choice experiments conducted under greenhouse conditions during August-September 2016 (24 °C mean temperature, natural daylight) at the Democritus University of Thrace. Potted cucumber plants (4 weeks old) were infested with 20 female thrips or 50 whiteflies per plant on the 2^nd^ (L2) leaf or left insect-free for 7 days in the greenhouse. To exclude effects of any insect cues on treated leaves, L2 was cut with a sterilized razor blade from both the infested and control plants on day 8 (Fig. 1). For each choice experiment, plants were placed in pairs (each plant representing a different infestation type, Fig. 1) inside thrips-proof insect cages (100 × 100 × 100 cm) at a distance of 60 cm. Depending on the experiment, 50 thrips or 100 whitefly females enclosed in glass tubes (50 ml) were released at once in the centre through the sleeve without opening the cage. Plants were carefully inspected after 2, 4, 6 and 24 hours and the number of insects on each plant was recorded. Experiments were repeated in two time blocks with 4 experimental replicates (pairs of plants) per time point for each species.

To assess the overall impact of choices available on preference the proportions of thrips or whiteflies making a choice among the two plants of each combination at each time point were analysed with a generalized linear mixed model (GLMM) with a binomial error distribution. In these models, choice (i.e. combination of plants of different type of infestation) and time were specified as fixed factors and pair of plants nested within block (time replicate) as random factor.

In addition, for each choice experiment and insect species the distribution of the insects over the two plants (representing a different infestation type) through time was compared using a GLMM with a Poisson error distribution for the numbers of thrips and whiteflies that were recaptured on each plant. In these models, choice infestation type and time were specified as fixed factors and plant (experimental unit) nested within pair of plants and block (time replicate) as random factor. Differences in the number of whiteflies or thrips recaptured on each plant would result in a significant effect of type of infestation, whereas differential change (increase or reduction) of this number over time would result in a significant interaction of type of infestation with time.

Significances of model terms were calculated using Wald’s Type II Chisq test (function ‘Anova’) in the R package ‘car’^27^.

### Data availability

The datasets generated and analysed during the current study are available from the corresponding author upon reasonable request.

## Discussion

Plant-mediated effects are recognized as major forces in shaping herbivore interactions^2,13,14^. In this study, we explored the ways omnivores and pure herbivores interact via the plant and revealed a hidden player in whitefly-thrips interactions. Our data show that whitefly-thrips interactions result in bi-directional competition that is differentially expressed against the two species. Moreover, performance and preference were shown to be affected suggesting that physiological and behavioural mechanisms that are not mutually exclusive are involved in competitive interactions among whiteflies and thrips.

Whiteflies and thrips were shown to be differentially affected via the plant in interspecific interactions; adult whiteflies negatively affected juvenile thrips survival (Fig. 4b, Table 1), whereas thrips impacted whitefly oviposition (Fig. 3a,b, Table 1). These responses were recorded on leaf discs that were cut from leaves systemically connected with the treated leaf for the development experiments, and in local and systemic leaves of whole plants for the oviposition experiments. The systemic expression of these effects suggests the involvement of transportable cues most possibly related to the plant’s response to the phytophagy and/or oviposition of the adults (prior infestation) and not to insect-derived kairomones. In addition, it highlights the ability of both insects to alter the shared host plant to their benefit not only when they are temporally but also when spatially separated. Nevertheless, thrips oviposition was not affected on whitefly-infested plants (Fig. 5a,b, Table 1) and whitefly juveniles developed to adulthood at the same rate on thrips-infested plants as when developing on clean plants (Fig. 2a,b, Table 1). As plant-mediated indirect interactions between different pests have been demonstrated to be impacted by the feeding strategies of the interacting organisms^28,29^, the differential responses recorded in our study may be attributed to the feeding modes of whiteflies and thrips that result in the elicitation of distinct signaling pathways that are known to interact.

Whiteflies are phloem-feeders that cause little damage to the plant tissue during their ‘stealthy’ feeding^15,30,31^. They are known to induce the transcription of salicylic acid (SA)-related genes while suppressing jasmonic acid (JA)-related responses in various plants^30,32–34^. On other hand, thrips are cell-content feeders that induce JA-mediated defences and *Arabidopsis* and tomato resistance against thrips has been attributed to the negative effects of JA-related defences on thrips performance and behaviour^15,35–38^. In our study however, thrips laid a similar number of eggs in all treatments (Fig. 5a,b, Table 1), suggesting that no defences were effective against adult thrips on cucumber plants. In contrast, thrips larvae were shown to suffer increased mortality on leaf discs systemically connected to whitefly-infested leaves. This might be caused by the ability of whiteflies to gather assimilates to their feeding sites by manipulating the sink source flow^39,40^. In that case, thrips larvae fed on nutritionally inferior leaf tissue that was systemically connected to the whitefly sinks. However, this mechanism should not be effective against whiteflies for which we recorded facilitation on leaf tissue systemically connected to whitefly-infested leaves.

Little is known about the effects of JA-defences against whiteflies. In general, whiteflies are believed to be JA-sensitive: in previous studies whitefly preference for oviposition was shown to be negatively affected by JA-defences (however, without affecting oviposition) and nymph development was accelerated on plants impaired in JA; nevertheless, development on JA-enhanced tomato plants was delayed compared to control plants^33,41^. Our data show that whitefly oviposition was reduced (Fig. 3a,b, Table 1), whereas nymph development was not affected on thrips-infested plants (Fig. 2a,b, Table 1). An explanation might be that whiteflies require a relatively long time to reach adulthood (approx. 15 days) and, unlike adults, whitefly nymphs continuously feed on the same spot throughout development. This might have masked the effects of otherwise effectual JA-responses that were induced during the four days of thrips infestation. Egg retention by adult whiteflies could explain the lower number of eggs found on thrips-infested plants but this needs to be further explored.

Whiteflies and thrips were shown to be differentially affected via the plant in intraspecific interactions. The performance of whiteflies was better on plants infested with conspecifics (Figs 2 and 3), whereas thrips performed equally well on thrips-infested and clean plants (Figs 4 and 5) suggesting that no effectual defences against thrips were induced in these experiments. Whiteflies ability to manipulate the sink-source flow and/or to suppress effective JA-related defences^39,40^ may explain the faster development and increased oviposition recorded on whitefly-infested plants in our study. Early arrival on a plant may be an effective strategy of insects to avoid competition with other herbivores^42,43^. In that case, facilitation shown in our study would allow whiteflies to precede in their population build-up and potentially avoid, win or withstand competition with thrips and other competitors.

For this strategy to be effective whiteflies should be able to discriminate plants already infested with conspecifics. Our data clearly show that whiteflies strongly preferred whitefly-infested over thrips-infested or clean plants (Fig. 6b,c, Table 3). To exclude insect derived cues that could influence insect choices in our study infested leaves were removed from all plants used in the choice greenhouse experiments. Therefore, we argue that volatiles released in response to prior feeding may have contributed to the accumulation of most whiteflies on these plants. The fact however that whiteflies failed to discriminate thrips-infested over clean plants (Fig. 6a, Table 3) may suggest that the impact of thrips infestation on plant volatile emission is lower, or that whiteflies are more sensitive to SA-activation (whitefly-infested plants that are strongly attractive) than to JA-activation (thrips-infested plants that are not strongly repulsive). Furthermore, it may suggest that an exposure (learning) mechanism is required for efficient whitefly decision-making. Adult females of the whitefly *Bemisia tabaci* were shown to learn to avoid plants with predatory mites whose previous and present diet consisted of whitefly eggs and nymphs^44^ but not when their present diet consisted of non-whitefly food (e.g. pollen)^45^. Similarly, to avoid intraguild predation and reduced oviposition, whiteflies would benefit from being able to discriminate thrips-infested plants over clean plants. In our study, whiteflies came from a whitefly rearing and thus were ‘experienced’ in whitefly-infested plants but ‘inexperienced’ in thrips-infested plants where thrips had access to only plant food (and not whitefly prey). Moreover, time for whiteflies to learn may far precede the duration of these experiments (48 hours) suggesting that whitefly preference needs to be further studied by including experienced individuals to the experimental setup. The role of volatile emission in attracting whiteflies on whitefly- and thrips-infested plants needs to be further studied in olfactometer behavioural assays coupled with headspace volatile analyses.

Despite being ‘experienced’ in thrips, released thrips did not discriminate among thrips-infested and whitefly-infested or clean plants (Fig. 6d,f, Table 3). In addition, they showed no preference for whitefly-infested over clean plants (Fig. e, Table 3). Thrips juveniles were shown to be negatively affected by whiteflies (Fig. 4a,b), but adult fecundity was similar on all plants (Fig. 5a,b). Therefore, one might think it would be of thrips benefit to avoid whitefly-infested plants. Most importantly however, thrips would benefit on residing on the same plants as their prey (whitefly eggs and nymphs)^5^. Similarly, thrips showed no preference nor avoidance for spider-mite infested plants^46^. The inability of thrips to detect whitefly-infested plants might be explained by the impact of whiteflies on plant volatile emission being low (but still detectable by the whiteflies), and/or lack of conditioning to whiteflies. On the other hand, thrips performed equally well on thrips-infested and clean plants (Fig. 5a,b) and therefore would not gain any benefit by being able to choose among them. As with whitefly preference experiments, headspace volatiles analyses would cast light on the role of volatiles in shaping these interactions.

We conclude that physiological and behavioural mechanisms are involved in omnivore-herbivore plant-mediated interactions. Whiteflies and thrips engage in competitive interactions via the plant that are differentially expressed against each species in response to their phytophagy and/or oviposition. Whiteflies were shown to display anti-predator defences against thrips by impacting juvenile thrips (this study) or via their stealthy presence and ability to manipulate/withstand plant responses^15,30,31,33^. Thrips on the other hand, are important intraguild predators^4–6^, but were also shown to affect whitefly performance (oviposition) via the plant (this study). Thus, plant-mediated competition among whiteflies and thrips can be bi-directional and, importantly may affect/strengthen the outcome of predator-mediated competition^11,12^ that is especially relevant with biological control. As recently shown however, the outcomes of indirect plant-mediated interactions are strongly dependent on the sequence of each species arrival^42,43^. It would therefore be relevant to investigate whitefly-thrips interactions in this context or when both species are simultaneously attacking plants. Further studies could cast light on the regulatory mechanisms involved to these competitive interactions.

Considering plant-mediated effects could help explain omnivore/herbivore population dynamics in the field and enhance our understanding of the ecology of the complex relationships among plants, herbivores and predators. This will potentially allow us to enhance sustainability in pest control.
